# Genetics and biology of *Anastrepha fraterculus*: research supporting the use of the sterile insect technique (SIT) to control this pest in Argentina

**DOI:** 10.1186/1471-2156-15-S2-S12

**Published:** 2014-12-01

**Authors:** Jorge L Cladera, Juan C Vilardi, Marianela Juri, Laura E Paulin, M Cecilia Giardini, Paula V Gómez Cendra, Diego F Segura, Silvia B Lanzavecchia

**Affiliations:** 1 Instituto Genética EA Favret, Instituto Nacional Tecnología Agropecuaria, 1686 Hurlingham, Provincia Buenos Aires, Argentina; 2 Departamento Ecología, Genética y Evolución, Facultad Ciencias Exactas y Naturales, Universidad Buenos Aires; 1428 Ciudad Autónoma Buenos Aires, Argentina; 3 Instituto de Ecología, Genética y Evolución de Buenos Aires, Consejo Nacional de Investigaciones Científicas y Técnicas, Buenos Aires, Argentina

## Abstract

Two species of true fruit flies (taxonomic family *Tephritidae*) are considered pests of fruit and vegetable production in Argentina: the cosmopolitan Mediterranean fruit fly (*Ceratitis capitata *Wiedemann) and the new world South American fruit fly (*Anastrepha fraterculus *Wiedemann). The distribution of these two species in Argentina overlaps north of the capital, Buenos Aires. Regarding the control of these two pests, the varied geographical fruit producing regions in Argentina are in different fly control situations. One part is under a programme using the sterile insect technique (SIT) for the eradication of *C. capitata*, because *A. fraterculus *is not present in this area. The application of the SIT to control *C. capitata *north of the present line with the possibility of *A. fraterculus *occupying the niche left vacant by *C. capitata *becomes a cause of much concern. Only initial steps have been taken to investigate the genetics and biology of *A. fraterculus*. Consequently, only fragmentary information has been recorded in the literature regarding the use of SIT to control this species. For these reasons, the research to develop a SIT protocol to control *A. fraterculus *is greatly needed. In recent years, research groups have been building a network in Argentina in order to address particular aspects of the development of the SIT for *Anastrepha fraterculus*. The problems being addressed by these groups include improvement of artificial diets, facilitation of insect mass rearing, radiation doses and conditions for insect sterilisation, basic knowledge supporting the development of males-only strains, reduction of male maturation time to facilitate releases, identification and isolation of chemical communication signals, and a good deal of population genetic studies. This paper is the product of a concerted effort to gather all this knowledge scattered in numerous and often hard-to-access reports and papers and summarize their basic conclusions in a single publication.

## Background

Two species of true fruit flies (taxonomic family Tephritidae) are considered pests of many fruits and vegetables in Argentina: the cosmopolitan and well known Mediterranean fruit fly (*Ceratitis capitata *Wiedemann), and the less conspicuous South American fruit fly (*Anastrepha fraterculus *Wiedemann). In this country the distribution of these two species overlaps from north of San Juan Province (parallels 30/31º S), in the extreme west, to the north part of Buenos Aires Province (parallels 34/35º S) in the east, and extends all the way to the northern border of Argentina. *A. fraterculus *is particularly present in the subtropical north-east (NEA) and north-west (NOA) regions [1], where the weather is warm and humid. These two regions are separated by the bio-geographical province of Chaco [2], a very arid region where *A. fraterculus *is normally absent (see references in: [3-5]).

Regarding the control of these two pests, the varied geographical fruit-producing regions in Argentina involve quite different situations. Patagonia and southern Cuyo are fruit fly free areas. Northern Cuyo (where *A. fraterculus *is not present) is under a pest management programme using the sterile insect technique (SIT) for the eradication of *C. capitata*. The main strategy to deal with the fruit fly problem in NOA is to follow quarantine protocols for the export of fruit (mainly lemon), whereas in NEA chemical control is applied and monitoring records are kept for both pests.

*Ceratitis capitata *is thoroughly known because of its wide prevalence in many places of the world. Regrettably, only initial steps have been taken to investigate the genetics and biology of *A. fraterculus*. As a consequence, a well-established protocol is available for the application of the SIT as a control method for *C. capitata*, but only fragmentary information has been recorded in the literature regarding the use of the SIT to control *A. fraterculus*. For this reason, research to develop a SIT protocol to control *A. fraterculus *is greatly needed.

In recent years, a research network has been building up in Argentina in order to address particular aspects of the development of the SIT for *A. fraterculus*. Besides two groups at Instituto Nacional de Tecnología Agropecuaria (INTA) in Castelar, Buenos Aires, scientific and technical studies are underway at the Universities of Buenos Aires (UBA) and Tucumán (UNT), the EEAOC (Estación Experimental Agroindustrial Obispo Colombres) in Tucumán, INTA Experimental Stations in Concordia (Entre Ríos) and San Pedro (Buenos Aires), besides laboratories at CNEA (Comisión Nacional de Energía Atómica) in Ezeiza (Buenos Aires) and from Instituto de Investigaciones Bioquímicas de Buenos Aires (IIBBA, Consejo Nacional de Investigaciones Científicas y Técnicas), and Planta Piloto de Procesos Industriales Microbiológicos (PROIMI, Consejo Nacional de Investigaciones Científicas y Técnicas) in Tucumán.

The problems being addressed by these groups include improvement of artificial diets and mass rearing, radiation doses and conditions for insect sterilisation, basic genetic knowledge supporting the development of males-only strains, reduction of male maturation time to facilitate releases, identification and isolation of chemical communication signals and population genetic studies. This paper is the product of a concerted effort to gather all this knowledge scattered in numerous, relatively inaccessible reports and papers and summarize their basic conclusions in one publication.

## Anastrepha fraterculus

The nominal species *A. fraterculus*, is a highly polyphagous pest reported to occur from southern United States (Texas) to Argentina [6,7], attacking over 80 species of plants along this range, including major fruit crops [8,9]. Its presence limits international trade because of quarantine regulations to avoid cross-border introductions [7]. This pest and *C. capitata *are the only fruit fly species of economic and quarantine importance reported in Argentina. These species are also economically important in large fruit production areas of Peru, Uruguay, and southern Brazil (see [10,11] and the present review). The development of technologies and strategies for control and/or eradication of both species simultaneously is of great interest. Furthermore, the application of the SIT to control *C. capitata *in overlapping areas with the possibility of *A. fraterculus *occupying the niche left vacant by *C. capitata *becomes a cause of much concern. Any effort to remove, suppress or exclude *A. fraterculus *from fruit producing regions should have a positive impact on local development and regional economies. In contrast, if no effective control method is developed against *A. fraterculus *in the near future, possible population expansions of this species might greatly reduce the benefits of *C. capitata *control in those areas of coexistence [10,11].

At present, the only control method available for *A. fraterculus *is the use of bait sprays. This represents a problem particularly in areas where it coexists with *C. capitata*. In such situations, the application of the SIT against *A. fraterculus *is a very attractive alternative ([11], see more ref. in [12]). At the South American regional level, research on the possibility of using the SIT to eradicate populations of *A. fraterculus *was initially reported in a workshop organised by the International Atomic Energy Agency (IAEA) at Viña del Mar in November 1996 [10], but prior to that, a number of investigations had already been advancing; especially at the University of São Paulo and Empresa Brasileira de Pesquisa Agropecuária (EMBRAPA), Rio Grande do Sul, in Brazil; and, in Argentina, at Instituto de Genética INTA, the University of Buenos Aires, and Centro de Investigaciones para la Regulación de Poblaciones de Organismos Nocivos (CIRPON-Tucumán) [6,13-16]. Furthermore, already in the 1970's, Peruvian researchers at La Molina facilities in Lima performed pioneering efforts in this direction producing a series of reports published in local journals [17]. However, a problem with this early research is that the taxonomic delimitation of the entities under analysis was not entirely clear.

In reference to the taxon *A. fraterculus*, many reports have compared flies from different sites or hosts (see references listed recently by Cáceres *et al*. [18]). Records show differences in morphology [19], karyotypes, isozymes [20], host preference [21], egg morphology [22], hybridisation [23], mitochondrial DNA [24], highly repetitive DNA [25], morphometrics [26], and mating compatibility [27]. Many authors have indicated that the nominal species, *A. fraterculus*, actually is a complex of species (for a first revision, see [7]; and for additional discussion, see [26,28,29]).

Resolution of this complex is of outmost importance. This problem is now being addressed by a multidisciplinary research project coordinated by FAO/IAEA. Specialists from Argentina, Brazil, Colombia, Czech Republic, Italy, Mexico, and USA met recently (August 2013) in Tucumán, Argentina, and agreed on the fact that *A. fraterculus *is composed of at least **seven **different biological entities: "Ongoing studies using different methodologies (DNA, morphology, cytology, sexual behaviour, and the chemical profile of male-emitted volatiles and cuticle extracts) confirm the existence of several of these species. This result is also supported by a comprehensive morphological study that incorporates collections from the whole region. Finally, a large number of mating crosses among various origins points towards the fact that the population differences are correlated with behavioural reproductive isolation. Research is ongoing to define species limits and their distribution, as well as to formally name these putative species. This will be critical for international trade and any SIT application (Insect and Pest Control Newsletter Nº82, January 2014, p.15). In fact, as mentioned by Silva and Barr [30], the delimitation and identification of a species or a complex of species is essential for basic and applied research and have far-reaching practical consequences, as is the SIT implementation. This characterisation needs to be achieved by studies about genetic, morphology and behaviour.

## Biology of *Anastrepha fraterculus*

For an efficient and effective application of the SIT we need an adequate knowledge of the biology of the pest species in general, and of the potential target populations in particular. The successful application of the SIT requires the ability to rear, sterilise and distribute sufficient insects to achieve a high sterile-to-wild insect ratio in the field, and also that the sterile males can successfully compete and mate with their wild counterparts after being mass-produced in an artificial environment, exposed to ionising radiation, densely packed and shipped to a distant facility, often immobilised, chilled, and ejected from flying aircraft [31]. The key biological aspects that determine the suitability of laboratory strains for SIT have been identified as: colonisation procedures and strain management, especially studies on insect nutrition, irradiation protocols, field dispersal and survival, field cage behaviour, and mating compatibility and competitiveness [31]. For *A. fraterculus s*ome of these aspects have been reviewed by Cáceres *et al*. under the umbrella of "quality management" [32]. We review here some aspects on *A. fraterculus *biology that may be necessary to apply the SIT on this species.

The literature on life history strategies of tephritid fruit flies, reviewed by Fletcher [33], reports for *A. fraterculus *that adults do not disperse long distances, live 3-4 months in the laboratory, spend unfavourable periods in the adult stage, mate away from host plants, feed on ripe fruit and produce 200-400 eggs per female (citing works of Malavasi and Morgante [34], and Malavasi *et al*. [35]). In recent years, Utgés [36] has evaluated the dispersal and the spatial distribution of *A. fraterculus*, which had received different pre-release diets. The average maximum distance reached was 150-160 m (according to records obtained for up to 8 days after release) and no differences were found among diets or between sexes. Larger capture densities were always near the releasing point and there was no apparent association with wind direction. The oviposition behaviour of *A. fraterculus *was first observed by Barros *et al*. [37] who described that after landing on the fruit, the female displays three stages: searching, puncturing (egg-laying) and dragging of the ovipositor over the fruit surface. Prokopy *et al*. [38] showed that in the process of dragging her ovipositor, the female deposits on the fruit an "oviposition-deterring pheromone".

Although an *A. fraterculus *strain was artificially reared in Peru as early as 1971 [39], the first report of artificial rearing was published by Salles in 1992 for *A. fraterculus *from Brazil [13]. This author tested the influence of photoperiod [14] and temperature [15] on development, finding that the life cycle may be completed between 20 and 25 ºC, but largest amounts of eggs are laid at 25 ºC. A more thorough work by Cardoso *et al*. [40] evaluated the effect of temperature on the reproductive potential, life span, and life expectancy. By 1996, four research groups (in Peru [41], Brazil [42], Argentina [43], and Colombia [44]) were rearing *A. fraterculus *and performing small scale experiments in laboratory. A preliminary protocol for mass rearing *A. fraterculus *in Argentina was first published by Jaldo *et al*. [45] and Vera *et al*. [12] improved this technique and tested quality control parameters on a medium size scale of production. The nutritional requirements of *A. fraterculus *comparing different diets containing sugar, protein or other nutrients, either simultaneously or alternating have been then extensively investigated [36,46-48]. The general conclusion is that adult flies are able to select the food according to their needs and, for rearing facilities, the strategy of offering sugar and protein in different feeders could lead to an optimal ratio in terms of maturation and survival.

The survival of laboratory-reared sterile insects must be investigated because, as mentioned above, the mass-rearing and sterilisation processes required by the SIT may cause loss of fitness [49-51]. Experiments in field cages performed in Argentina showed that laboratory-reared males (either irradiated or not) may have a similar or higher survival rate compared to wild ones [46]. Incidentally, survival in field cages is drastically shorter than under laboratory conditions [40,52]. This indicates that field cage represents a challenging environment simulating open field conditions useful for quality control tests for laboratory-reared males. The effect of nutrition on *A. fraterculus *survival in open field was also studied by Utgés [36]. A trend to a reduction in survival when adult flies had received a diet rich in protein was suggested; however, in open field experiments survival is inferred from trap captures (non-protein baits are not available for A*. fraterculus *yet), so the possibility that insects fed with proteins before release are less attracted to traps with protein baits cannot be ruled out as a potential bias in this study.

## Mating behaviour of *Anastrepha fraterculus*

Aluja and Norrbom stated that "The success of the SIT hinges on a deep understanding of behavioural mechanisms" [53]. Among them, the ability of sterile males to mate and transfer functional sperm to wild females is one of the key factors. This ability depends on the processes of sexual maturation, the courtship behaviour and the ability of released sterile males to modulate re-mating behaviour in females. All three aspects were investigated in *A. fraterculus *and are briefly reviewed here.

A long pre-copulatory period of adult males poses a problem in the practice of the SIT. When sterile flies are maintained at the fly handling facilities for several days before their release, the operational costs (food, space, and staff cf. [54]) considerably increase. Besides, holding adults may also lead to physical damage to the flies (cf. [55]), sometimes forcing the release of sexually immature flies, which are not able to compete with wild males.

**Figure 1:**
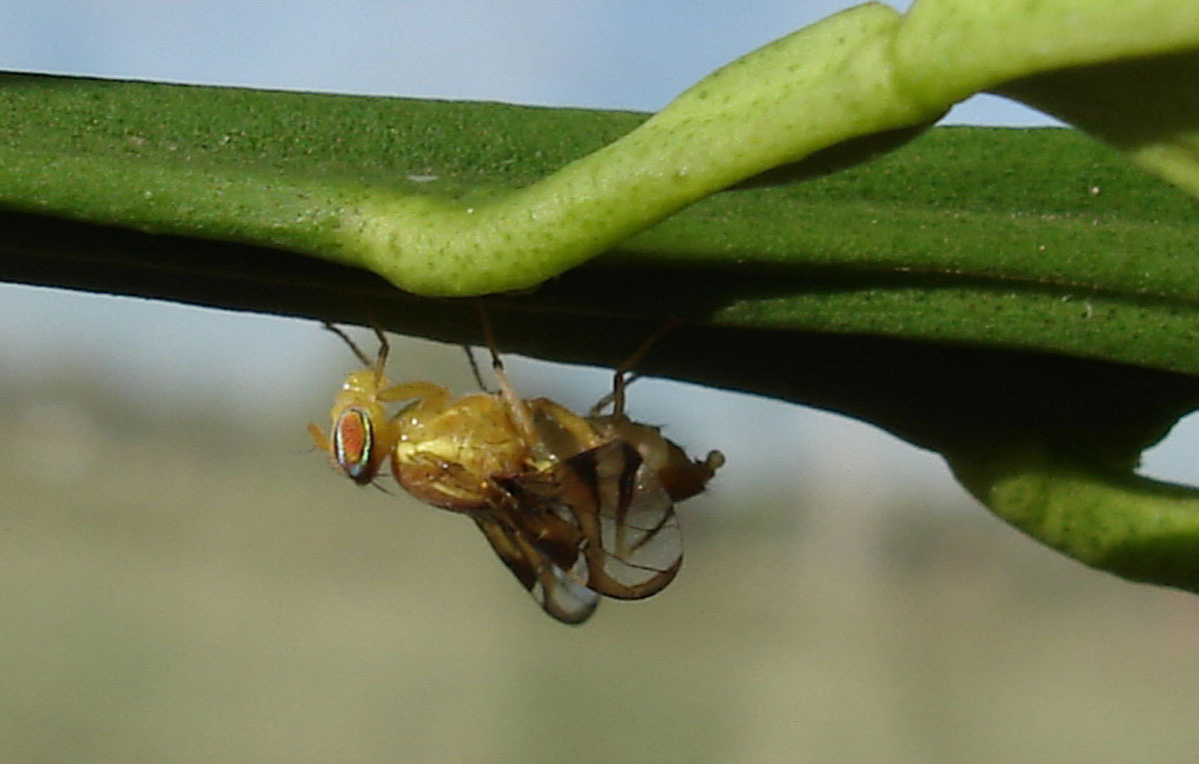
**Male *A. fraterculus***. Calling male expanding the abdominal pleura where the glands are located

As for other *Anastrepha *species, in *A. fraterculus*, sexual maturation is a slow process, so the male maturation problem has received a good deal of attention. Lima *et al*. [52] reported that males reach complete sexual maturation 8-9 days after emergence, and Salles found that some males start pheromone calling (males expand the abdominal pleura, where the salivary glands are located [56]; see Figure 1) at day 5 after adult emergence [42,57]. More recently, Segura *et al*. [58] have found high variability in the age that males need to reach to exhibit the pheromone calling (Figure 1) and be able to mate; the average is approximately 7 days after emergence, but some males start to mate with virgin females at day 4 and others need 10 days. Some evidence of the genetic control of this variability was found studying mutant strains of *A. fraterculus*. The Sexual maturation process was significantly faster in one strain than in the others and this trait was paternally inherited [59].

The juvenile hormone analogue methoprene (applied topically) shortens in ca. 3 days the pre-copula period of sterile *A. fraterculus *males under laboratory conditions [58]. The mating competitiveness in field cages of young methoprene-treated males was found very satisfactory [60-62]. It has been also found that methoprene did not accelerate maturation in females to the same extent, so a sort of "physiological sexing" results as by-product, which may help to increase the efficiency of the SIT against *A. fraterculus *[61]. The age of first calling also varies in response to the adult diet [36], so the implementation of methoprene acceleration would also require that flies receive sugar and protein before they are released [60,61]. Methoprene-treated males induce in wild females less "refractoriness" to re-mate than mature wild males [62]. Probably the juvenile hormone analogue accelerates the mating onset in *A. fraterculus *males but does not act as efficiently in the synthesis of males' accessory glands products, which modulate females re-mating behaviour, a hypothesis that needs evaluation.

Once sexually mature, the *A. fraterculus *male engages in complex sexual behaviours that may become critical in deciding success or failure of the SIT released male insects attempting to mate with females in the wild. These behaviours include, besides the above mentioned pheromone calling, "lekking", acoustic signals, increased motion activity and wing displays. These activities as well as the effects of rearing conditions on them are briefly reviewed here.

Lekking: To attract females and mate, the males of some species aggregate in a group denominated lek. In *A. fraterculus *each lek is integrated by 3-8 males [35] who increase their mating probability by investing more time in calling [63]. Some males may be calling outside the lek or alternating inside and outside [35], however, males that call always inside the lek mate more frequently than the others [63]. This stresses the importance of calling location in male mating success.

Courtship: Mating in *A. fraterculus *takes place mostly on the abaxial surface of those leaves that are more exposed to sun light, where the lek is usually located [35,64]. The diel pattern of calling activities differs in flies from different geographical origin [18,27,65]. In populations studied by Malavasi and co-workers in Brazil [35,64] and Petit-Marty *et al*. in Argentina [66,67], males start pheromone calling soon after sunrise and end before noon. The sequence of behaviours that leads to a successful mating was registered through video recordings by Gómez Cendra *et al*. [68]. Successful males generally reached copulation within 10 minutes after a female was in their proximity. Males that did not reach copulation exhibited some behavioural differences when compared with successful ones [68]. Vibratory cues produced during calling are also involved in *A. fraterculus *courtship. Differences in the calling song among different populations might also be related to the existence of pre-zygotic isolation barriers [64,69,70] (see below).

Chemical cues: *A. fraterculus *wild males attract females on the basis of chemical signals (sex pheromones), which may be important in mating success and compatibility between strains. The components of sex pheromones in extracts from salivary glands were studied by gas chromatography coupled with mass spectrometry [71]. These studies show that *A. fraterculus *pheromone is a complex mixture of several compounds that vary largely in their relative amounts. The role of each of these compounds in courtship remains to be assessed, but differences in chemical profiles among different populations have been used to postulate the existence of pre-zygotic isolation barriers [18,72].

Plant compounds: Plant compounds have been found to affect male courtship and, indirectly, male mating competitiveness in other Tephritidae species (cf. methyl-eugenol in some *Bactrocera *species or ginger root and orange essential oils in *C. capitata *[73]). Exposure to guava fruit volatiles does enhance male mating success in laboratory *A. fraterculus *[74]. This response has been suggested to be associated to α-copaene, a compound that has been known to strongly improve mating success in *C. capitata *and is present in low amount in guava fruit, although other compounds might also be involved.

**Figure 2:**
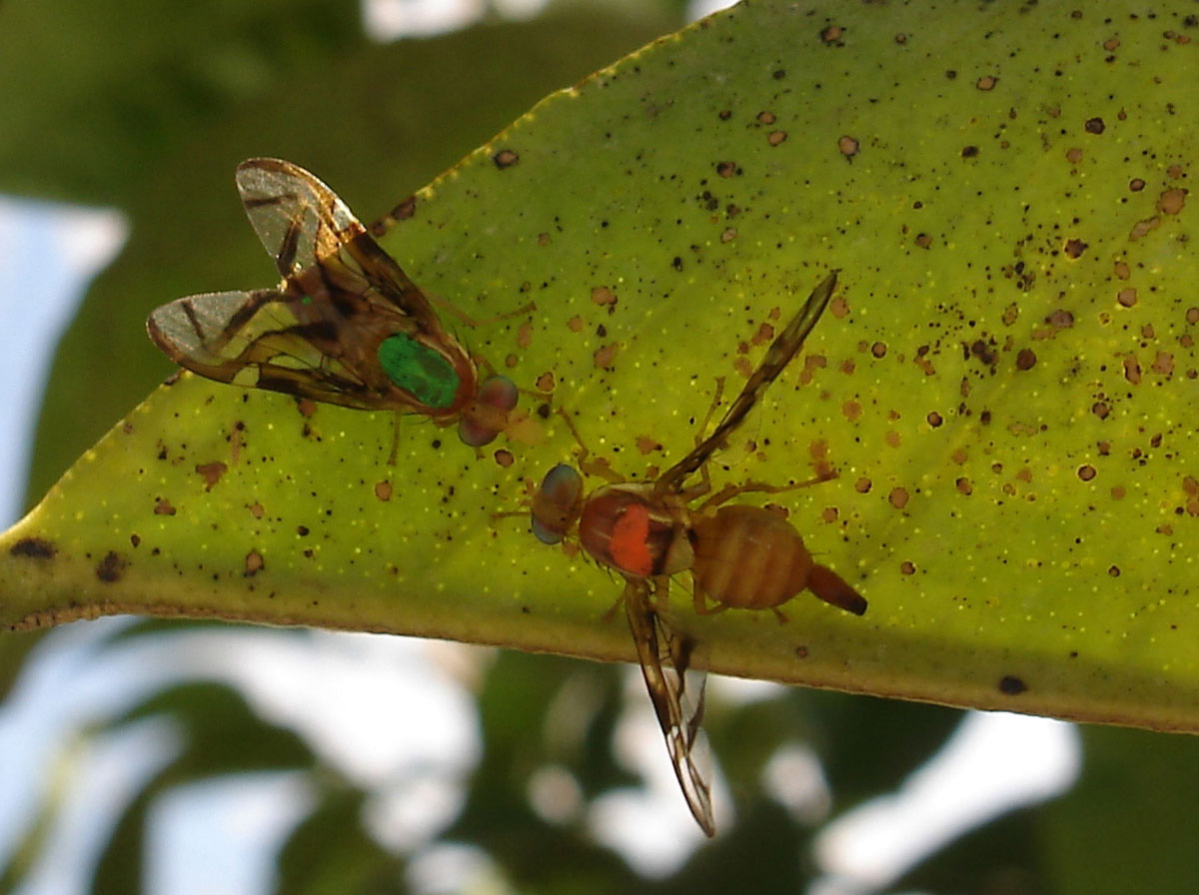
**Female *A. fraterculus***. Approaching a male in an attempt to copulate (dot of color paint on the thorax for identification of flies in a caged tree)

Sexual success: In the SIT the external morphology may be relevant to the mating success of released sterile males. Sciurano *et al*. [75] compared the multivariate phenotype between successful and unsuccessful males competing to copulate in caged trees ( see Figure 2). Specific traits, such as wing width and thorax length, were identified as most probable targets of sexual selection. Male mating success does not seem associated with size but rather to body shape. In fact, Segura *et al*. [63] found no relationship between body size and mating success or the ability of males to integrate into leks; however, the "face width" was found to be negatively associated to copula duration and positively associated with latency (the time between fly release into the cage and copulation), and the "eye length" was positively associated with copula duration and probability to mate. Artificial rearing may have a side effect on the multivariate phenotype of *A. fraterculus*. In general, lab flies are larger and show reduced variance in body size related traits compared to flies from the wild. Specifically, lab males have wider head, longer eye and narrower wing than wild males [5].

Re-mating: The female propensity to mate again after the first copula may be very relevant for the implementation of any SIT programme. The first record of *A. fraterculus *re-mating behaviour was performed by Lima *et al*. [52] in Brazil, but most studies on the frequency and other details of this phenomenon were conducted in Argentina by Abraham *et al*. [62,76-78]. The most important conclusions of this research are that a long-lasting first mating and a protein-rich male diet may reduce re-mating [77,78] and that female re-mating propensity seems to be associated to sperm depletion [76]. The amount of sperm stored by females is not affected by male irradiation, methoprene treatment, or protein intake. Interestingly for the application of the SIT, *A. fraterculus *from Argentina has a refractory period so long (16 to 19 days respectively for laboratory and wild females) as to be considered a functionally non-re-mating species [76].

## Irradiation dose for male sterilisation

The introduction of sterility in the females of the wild population is achieved following their mating with released males carrying dominant lethal mutations that have been induced in their sperm by radiation treatment [79]. The SIT can only be effective if the irradiated male is able to perform all the functions of a normal fertile insect, mainly it must carry fully functional sperm that succeeds in fertilising eggs and initiating their development [79]. Radiation biology studies are essential within the SIT framework [80]. In general, studies have focused on determining a radiation type (gamma or X) and a dose that guarantees full sterility of flies with no detrimental effect on males ability to inseminate wild fertile females. Environmental conditions (temperature, humidity, oxygen concentration, etc.) as well as the developmental stage, at which irradiation is carried out, have been also addressed for many species (see [81]). Specifically for Tephritidae the average sterilisation dose reported (based on 21 species) was 63Gy [80].

In this context, we review now some studies dealing with the effect of ionising radiation on sterilisation and development of *A. fraterculus*. On the Peruvian population a dose of 50 Gy induces sterility on males [17]. Female sterility is induced with doses of 30 Gy, but 60 Gy are needed to completely stop egg laying [17]. Allinghi *et al*. [82] showed that 60 Gy significantly reduce male fertility in flies from an Argentinian population, but complete male sterility requires a dose of 70 Gy, irrespectively of the age at which pupae were irradiated. In this study, 40 Gy were enough to suppress egg-laying in females.

Allinghi *et al*. also performed standard mating competitiveness tests in outdoor field cages (cf. IAEA [83]) where laboratory *A. fraterculus *males that were irradiated 48 h prior to emergence competed with fertile wild flies [84]. They showed that irradiation affects neither the mating competitiveness of sterile males or females, nor the latency to mate. A mild effect of radiation was found on mating duration, as fertile flies mated for a significantly longer period of time. Males irradiated at 40 Gy produced incomplete sterility on wild fertile females, but at a dose of 70 Gy (or higher) more than 99.8% sterility is induced [84]. The protocol proposed by Allinghi *et al*. [82,84] (gamma radiation at a dose of 70 Gy, 48 h preceding emergence) was further evaluated to test irradiation effects on other important aspects related to the effectiveness of the SIT. So far, radiation has shown no effect on survival [46], dispersal [36], sexual maturation [58], mating competitiveness after juvenile hormone treatment [60] or re-mating behaviour [62].

The effect of radiation on the development of the reproductive system of *A. fraterculus *was studied in male and female flies from Argentina [85]. Irradiated females showed a marked reduction on the growth rate of the ovaries, which turns evident by day 4 after emergence. On the other hand, testis size was not affected by irradiation; however, the organisation of the testis is noticeably affected: the growth zone is reduced in size, spermatids are difficult to detect and become round-shaped (losing their normal triangular shape), the zone where sperm remains as a bundle becomes larger and, concurrently, the zone with free sperm is smaller [85]. These findings have practical implications for the SIT because the differences in morphology and/or structure between sterile and fertile males can be used as a diagnostic tool to differentiate wild and mass-released laboratory male flies (when the fluorescent dye used to mark laboratory flies is not detectable). This diagnostic method arises as a by-product of sterilising flies with gamma radiation.

The current knowledge on radiation biology of *A. fraterculus *provides a good starting point for any control programme focused on this species. Nonetheless, the fact that the same radiation dose can affect flies in very different ways according to several biotic and abiotic parameters makes it reasonable to caution that assays, as those performed for the local morphotype in Argentina, should be replicated by other control programmes.

Another important venue of information that needs further research is the replacement of gamma irradiators by X-ray irradiators [86], as an alternative to deal with the difficulty to move radioisotopes across countries and the discontinuing in production of the Gamma Cell. (However, there are still some unsolved problems with X-ray irradiators: see IAEA Insect Pest Control Newsletter 81, July 2013). In any case, experimental approaches like the one followed by Bachmann [87], in which gamma rays and X-rays were compared will surely provide valuable information to support the future use of either type of ray.

## Chromosomes of *Anastrepha fraterculus*

Chromosomal studies are important from different points of view. Cytological studies may provide critical information to identify cryptic species and detect polymorphisms, which may help to solve the *A. fraterculus *complex. A deep karyotypic analysis and a description of polytene chromosomes in Dipterans are valuable tools for identifying chromosome translocations (either spontaneous or induced) that can be used to develop genetic sexing strains.

The original description (1958) of chromosomes in *A. fraterculus *was performed by Mendes for a Brazilian population [88]. He reported that the chromosomal constitution of *A. fraterculus *is 10A+XX (females) and 10A+XY (males), with terminal centromere localisations for all chromosomes, and somatic pairing between homologous [88]. In 1962, Bush studied the metaphase chromosomes of *A. fraterculus *from Mexico, where the heteromorphic (sex) chromosome pair was not present [89]. To explain the difference from Mendes result, Bush advanced the hypothesis that he was "more likely dealing with a sibling species" [89]. Later, in 1987, again in Brazil, Solferini and Morgante [90] confirmed the diploid number (2n = 12) and the distinction of X and Y sex chromosomes. Among the five pairs of acrocentric autosomes, one of them is described as "characteristically larger" than the others. These authors reported a polymorphism concerning the sex chromosomes and concluded that some of the morphs represent members of a complex of cryptic species. Also in Brazil, in 1996, Selivon [91] separated samples of *A. fraterculus *from different places and hosts in two groups according to their isozyme patterns and showed that the two groups exhibited differences in the size of X and Y chromosomes. Further studies by the same author indicated that the two groups actually represent two cryptic species, naming them as *A. sp*.1 affine *fraterculus *and *A. sp*.2 affine *fraterculus *[28]. Also a probable third species in the *A. fraterculus *complex present in coastal locations of Brazil [92] and a fourth one from Ecuador [92] were described. Recently, Goday *et al*. [29] have analysed the heterochromatin organisation in mitotic chromosomes of eight *Anastrepha *species, including the four putative members of the *fraterculus *complex, using fluorescent staining and C-banding. The supposed distribution of one of them, "sp1", includes Argentina [29].

**Figure 3:**
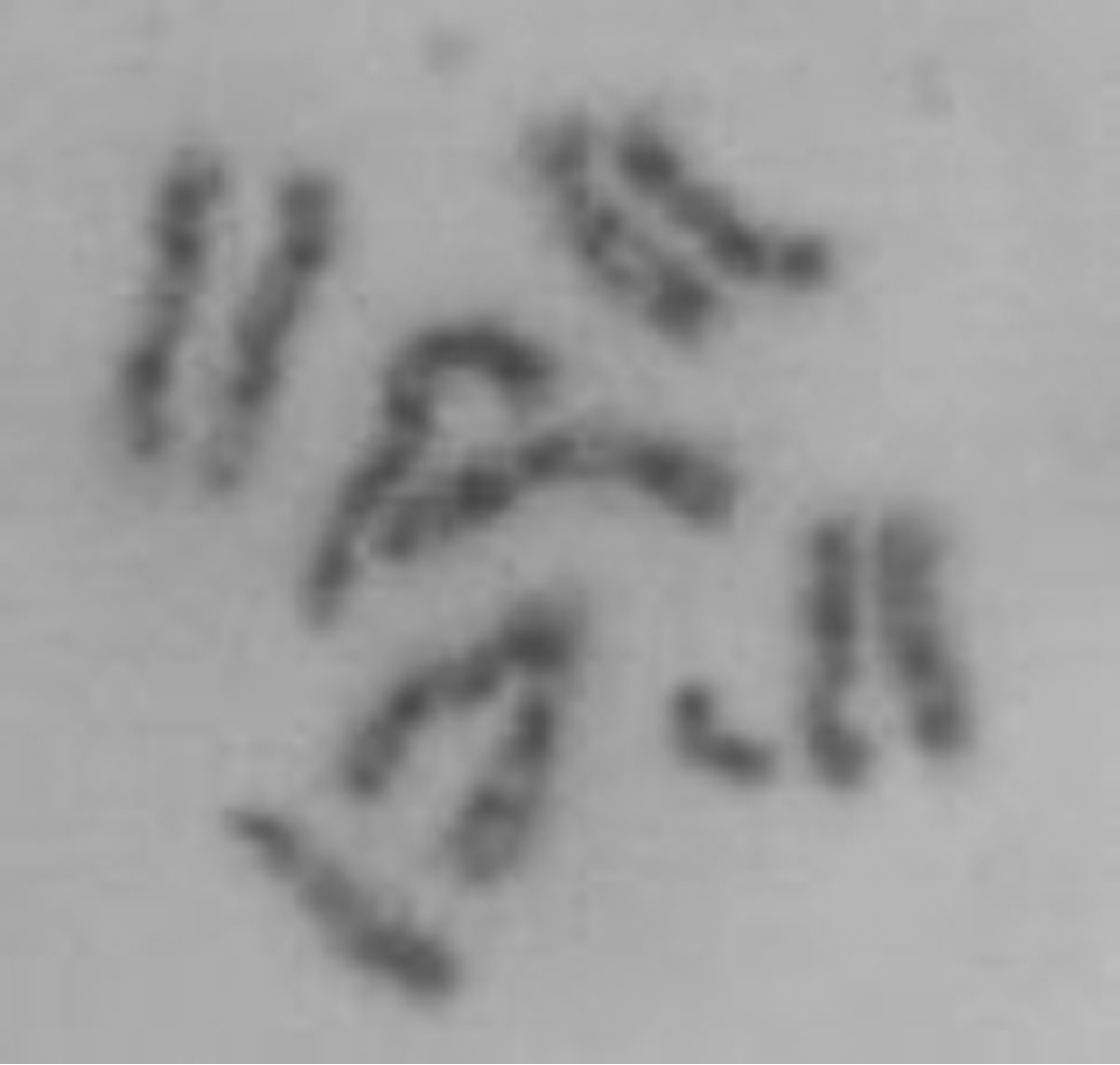
**Mitotic chromosomes of *A. fraterculus***. Somatic pairing of the five pairs of acrocentric autosomes, with the longer chromosome 2 in the center; the acrocentric X chromosome and the small sub metacentric Y chromosome are not paired

In Argentina, the initial report (in 1999 by Lifschitz *et al*.) of chromosomes of local populations of *A. fraterculus *[93] described a karyotype composed of five pairs of homomorphic and acrocentric autosomes, an acrocentric X chromosome and a small submetacentric Y chromosome whose length is approximately 2/3 of the X length (please refer to Figure 3). The autosomes were reported as almost indistinguishable from each other except for the longer chromosome 2 (Figure 3). C banding revealed two terminal blocks of heterochromatin in the X chromosome. The Y chromosome shows a pericentric C band. A detailed C-banding ideogram and an N-banding ideogram of this karyotype were published in 2003 [94]. This karyotype was the prevalent in all the samples studied in Argentina. However, occasional polymorphism of the sex chromosomes was present [94] (see also [95]). Four morphological variants of the Y chromosome and five variants of the X chromosome were reported to be present at low frequency in different samples of several localities in Argentina [93,96]. Laboratory strains carrying two different X and four different Y chromosomes, as well as two configurations for one of the autosomes, were later isolated. The viability and the survival for several generations of these strains as well as of individuals with different hybrid configurations [94] proved that the different chromosomal configurations found in the *A. fraterculus *populations studied in Argentina do not represent any indication of reproductively separated species, but rather, mere examples of chromosome polymorphisms [95].

A study of mitotic metaphase in hybrids between *A. fraterculus *from Argentina and Peru was included by Cáceres *et al*. [18] in a multidisciplinary approach to investigate isolation between cryptic species of this complex. These two strains could be differentiated by the size and morphology of their mitotic sex chromosomes; the Argentinian strain had a large X-chromosome (XA) with two prominent C-bands located at two tips, one band being larger than the other. The Argentinian Y-chromosome (YA) was smaller than the XA and also shows two C-bands, one on the proximal tip and the other in the sub-median region. In the Peruvian strain, both X- and Y-chromosomes were large and similar in size (XP and YP respectively). In the hybrid strain, some of the expected sex chromosome cytotypes (XAXP, XPXP, XAYA, XPYA, XAXA) were observed, as well as larvae with 13 chromosomes, either XXX or XXY, both with XA chromosomes [18].

The existence of giant (polytene) chromosomes present in the salivary glands of *A. fraterculus *was already reported in 1958 by Mendes [88], but it was not until 2009 when Giardini *et al*. [97] provided the first pictures of the polytene chromosomes of *A. fraterculus*. They identified each chromosome on the basis of constant morphological structures (landmarks) and specific features (e.g., puffing pattern). The authors also performed an approximation to the linear map of polytene chromosomes following the customary labelling system. A simultaneous analysis of mitotic and polytene nuclei carried out to determine the sex of the larva showed that neither the number of polytene chromosomes nor their banding patterns differentiate males from females, indicating that sex chromosomes do not form polytene chromosomes in *A. fraterculus *[97].

Also the polytene chromosomes were observed in the study mentioned above on hybrids between *A. fraterculus *from Argentina and Peru [18]. The banding patterns of the polytene chromosomes of the two parental strains were very similar, especially at the chromosome ends. The strain from Argentina showed very little polymorphism, whereas the Peruvian strain showed partial asynapsis and many inversions. The hybrids between strains (generations F1 and F2) confirmed significant similarities between both banding patterns as well as ample asynapsis along the chromosome complement, notably in almost all chromosome ends. An extensive asynapsis similar to that observed in the F1 and F2 hybrids was present even in the 14th and 20th generations of the follow up hybrid strain indicating that this level of incompatibility between Argentinian and Peruvian strains is maintained across generations [18].

## Population genetics of *Anastrepha fraterculus*

Studies on population genetics of insect pests provide valuable information to on-going control actions and also for programmes that are under development and evaluation, as is the case of the SIT for *A. fraterculus *in Argentina. The research about the distribution of genetic variability in wild populations, their colonisation patterns and phylo-geography are important to understand biological related problems for the control of insect pest species. In addition, we need the genetic characterisation of laboratory populations and mass rearing strains used for experimental research and for the SIT, not only for the genetic identification of lab strains, but also for their differentiation from wild populations. This information about the insect pests is helpful to give a complete landscape of genetics and ecology of the species to be used by control programmes to develop or improve the monitoring of released insects. The essential tools in all these studies are the protein and molecular markers, which are going to be reviewed here.

Initial studies conducted by Morgante *et al*. in 1980 on *A. fraterculus *population from Brazil [20] reported larger isozymes variation within this taxon than among other species of the genus. They postulated the existence of four morphologically indistinguishable groups within Brazilian *A. fraterculus*, with major differences between the northeastern and the southern populations. Allozyme frequency differences among Brazilian samples were not associated to the host plant [35,98]. Samples from northeastern Brazil were grouped by Steck [99] with others from coastal Venezuela, Central America, and Mexico; whereas southern Brazil samples were grouped with Andean Venezuela, and Peru [99]. The restriction fragment length polymorphism of a region of mitochondrial DNA amplified by PCR (mtDNA PCR+RFLP) was used to compare populations from Venezuela and Brazil, showing significant differences between lowland and Andean population. Both isozyme and mtDNA PCR+RFLP patterns confirmed a limited gene flow between southern and northern populations from Brazil [100]. Using sequences of the large subunit ribosomal DNA (16S rDNA) of the mtDNA McPheron *et al*. [101] also found that samples of *A. fraterculus *from Brazil clustered separately from samples from Venezuela. Other authors, like Smith-Caldas *et al*. [24] studied the mtDNA variation using Cytochrome Oxidase (COI) gene sequences. In the nominal species *A. fraterculus*, they distinguished 2 groups: 1) Andean populations that are separated from lowland Venezuelan populations and 2) Southern Brazilian population that is clustered together with the only population from Argentina that was included in the analysis.

In Argentina, although some *A. fraterculus *RAPD markers were reported in 1996 by Sonvico *et al*. [16] and isozymes were used in 1999 by Alberti *et al*. [102] in a genetic structure study (see below), the first extensive population genetic study of *A. fraterculus *was reported in 2002 by Alberti *et al*. [3]. Eight isozyme loci applied to nine populations from Argentina and restriction patterns of a PCR amplified mtDNA fragment (16S rDNA), studied in 11 Argentinian and one southern Brazilian populations, allowed these authors to arrive to the conclusion that all of these populations belong to the same species. Later Alberti *et al*. [103] sequenced a 417 bp fragment of the mtDNA COI gene and found no correlation between haplotypes and the geographic distribution in Argentina, finding new evidence against the presence of more than one species in the surveyed territory.

At a finer scale, a population structure study from Argentina compared the variation within and between fruits in flies emerging from guava collected at Yuto, in the northwest of Argentina [102]. The frequency of homozygote individuals was high, suggesting the existence of groups with a variable degree of genetic diversity, an unexpected genetic structure [102]. Recently the internal structure of a population in Tucumán was analysed applying ISSR (inter simple sequence repeats markers, developed at the University of Buenos Aires by Paulin *et al*. [104]). The variation within and among samples derived from different hosts was evaluated in relation to the chemical composition of these hosts by Oroño *et al*. [105]. The adaptation to plant chemistry appears to produce population differentiation. Besides, the differentiation is stronger between populations exploiting sympatric synchronic hosts differing in chemical composition than between populations that exploit hosts fruiting in succession [105].

Hopefully microsatellite markers, recently developed for *A. fraterculus *by Lanzavecchia *et al*. [106], may reveal higher levels of polymorphism in populations of this species than any other molecular tool so far available. About 140 regions analysed and 14 microsatellite loci selected already revealed remarkably high genetic variability in the four populations (two wild and two lab strains) used to test the markers in Argentina. These markers may also be used to study the genetic changes suffered by a wild population of *A. fraterculus *during the process of introduction into artificial rearing. Scannapieco *et al*. [107] demonstrated loss of genetic variability across the first few generations during the domestication process, similarly as described for other Tephritidae species [108,109]. These new microsatellite markers are currently being applied in our laboratory to the analysis of the genetic diversity in wild *A. fraterculus *populations from Argentina and Brazil.

We showed here some genetic and molecular tools used to characterise *A. fraterculus *at species and intra species levels with emphasis on information recorded from specific geographic regions in Argentina. One important challenge for the future will be to perform wider studies and careful analysis of population genetics with application of standardised methodology and the development of common DNA markers available to all researchers in the Americas to facilitate comparisons across borders. This knowledge would ensure having a complete picture of *A. fraterculus *genetics, information needed for the development and implementation of the SIT in this species.

## *Anastrepha fraterculus *isolation barriers

In relation to the successful implementation of a SIT programme to control *A. fraterculus *in South America, the problem requiring most urgent attention lies in the existence of cryptic species. Whitten and Mahon [110] clearly explain this situation: If we are dealing with a group of distinct species or even subspecies with limited interbreeding, each taxon must be treated separately for the SIT, since sterile males must be competitive with the field males in seeking female mates; the situation of the cryptic species could be even worse if the mating barriers are undetected because of the lack of relevant biological knowledge [110]. For this reason, the existence of at least seven cryptic species within the "*Anastrepha fraterculus *complex" [111] has become an incentive for the research on the existence of reproductive barriers and isolation mechanisms, as well as the degree of gene flow among them.

Pre-zygotic as well as post-zygotic isolation mechanisms among cryptic species of the *A. fraterculus *complex have been described. For instance, *A. fraterculus *sp. 1 and *A. fraterculus *sp. 2 from Brazil were crossed by Selivon *et al*. obtaining some reduction in the F1 egg hatch and sex ratio distortion [112]. In a larger experiment (including populations from Argentina, Brazil, Colombia and Peru), Vera *et al*. showed pre-mating isolation between flies from Peru and the other three populations, as well as between flies from Piracicaba (São Paulo, Brazil) and Argentina [27]. Moreover, flies from these two latter origins mate preferentially early in the morning, while Colombian flies mate late in the afternoon, and Peruvian flies mate preferentially around noon [27]. High levels of mating isolation were also found among Mexican, Peruvian and the Brazilian-1 morphotypes [113]. Here, the differences in morphology and genetics were correlated with the existence of limited gene flow, and post-zygotic mechanisms were also detected; however, their relative contribution to reproductive isolation was lower than pre-zygotic barriers [113].

The mechanisms behind the pre- and post-zygotic isolation barriers in the *A. fraterculus *complex are not well understood. In the previously mentioned study of hybrids between strains from Argentina and Peru [18], Cáceres *et al*. have found differences both in quality and quantity of certain parental pheromone compounds. Hybrid males' pheromone has been found to be a mix of the parental pheromones [18,114]. Interestingly, parental females did not discriminate between the males of their own strain and hybrid males [18], but hybrid females showed a marked preference for hybrid males [114]. In the chromosomes section, we have already mentioned extensive asynapsis in this hybrid between the Argentinian and Peruvian strains, suggesting significant genetic differentiation [18].

Petit Marty *et al*. [66,67] confronted *A. fraterculus *flies from extreme regions (NOA and NEA) inside Argentina. The authors determined the frequency of homotypic and heterotypic crosses in a large experiment under field cage conditions. No evidence of sexual incompatibility was found, either pre-zygotic [67] or post-zygotic [66]. These studies confirmed the presence of a single *A. fraterculus *biological entity in Argentina. In an attempt to delimit the boundaries of this morphotype that inhabits Argentina and extends to southern Brazil, Rull *et al*. [113] carried out pre- and post-zygotic tests, including two populations from Rio Grande do Sul, Brazil (Vacaria and Pelotas) and one population from Argentina (Tucumán), and found no evidence of sexual isolation among these populations, making a valuable contribution to the definition of the area occupied by this morphotype.

## The sterile insect technique and *Anastrepha fraterculus*

The idea of releasing insects of pest species to introduce sterility into wild populations, and thus control them, goes historically back to the 1930s and 1940s (see [115] for a review). The SIT is "a method of pest control using area-wide inundative release of sterile insects to reduce reproduction in a field population of the same species" [116]. Essentially, the SIT involves rearing a very large number of target species individuals, exposing them to ionising radiation (or chemosterilants) to induce sexual sterility and then releasing them into the target population where the sterile males mate with wild females preventing them from reproducing.

There are technical requirements as well as management and logistical prerequisites for a SIT programme to succeed [117]. The technical requirements are: availability of baseline data to develop an appropriate strategy, adequate competitiveness of the sterile males, mating compatibility between the strain used for release and the target population, assurance and persistence of the quality of the released strain and sound monitoring [117]. The management / logistical prerequisites are: commitment of all stakeholders, enough resources (funding, manpower and institutional capacity), flexible and independent management structure with dedicated full time staff, independent programme reviews, continuity in the implementation of critical programme components, data analysis plus feedback mechanisms, and public education and awareness [117]. For the existence of an *A. fraterculus *SIT programme in Argentina, we must say that presently most of the management prerequisites are still missing. In contrast, nearly all technical requirements have been fulfilled or are about to be.

The different aspects of the SIT have been thoroughly gathered in the book by Dyck, Hendrichs and Robinson [118]. There, the following technical components of the SIT are reviewed : 1) population and behavioural ecology, 2) mass rearing of the insect, 3) sterilisation with radiation, 4) quality of the sterile insect, 5) processes of supply, emergence and release, 6) monitoring of sterile and wild insects, and 7) a procedure for declaring the pest free status. In connection with the development of the SIT for *A. fraterculus *in Argentina most of these issues have been treated in the preceding sections; so we quickly retrieve them here in the aforementioned context.

Several genetic aspects of *A. fraterculus *populations from Argentina have been investigated [3,102,103,105,106], dispersal distances have been estimated [33-36] and the components of a successful sexual competitiveness dissected [5,63,75]. Estimating the fluctuations in the target population, the number of sterile males to be released, the density and the mortality rates are recommended by Ito and Yamamura [119]. These estimations for *A. fraterculus *are still missing.

Artificial-rearing of *A. fraterculus *has been investigated [12,13,45], the research on diet and nutritional requirements has also advanced [48]. But the up-scaling process to reach the production levels needed for the SIT has not been focus of research so far. Some of the issues that should be addressed are: facility design, environmental concerns, strain management, automation, sex separation, marking and storage [120].

Sterilisation with radiation of the *A. fraterculus *present in Argentina has been obtained [82,84,85]; the absorbed dose ensuring that treated insects are sufficiently sterile and yet able to compete for mates with wild insects is 70 Gy, applied 48 h before adult emerge. It is known that the oxygen level during irradiation influences both the absorbed dose required for sterilisation and the viability of irradiated insects [80]. However, irradiation in anoxia has not been investigated in this system up to now.

Quality, in terms of the ability of the insect to survive, interact with its environment, locate, mate and fertilise females of the target population has been tested for artificially reared *A. fraterculus *in field-cage tests under semi-natural conditions, where sterile males have to compete with wild males for wild females [36,46,58,60,62]. However, a compartmentalised system of bioassays for quality parameters assessment in the factory (cf. [121]) has not been completed yet.

Supply, emergence and release processes of the SIT for *A. fraterculus *were not investigated so far. Dowel, Worley and Gomes [122] recommend rearing insects in modules because this system offers flexibility and increases safety, compared to housing all the rearing process in a single building. Alternatively insects can be produced from purchased eggs or, adult insects may be obtained from specialised satellite facilities [122]. The release of sterile insects may be via static-release receptacles, ground-release systems or from the air, but aircraft guided by GPS and computer controlled release of chilled sterile insects has proved to be most efficient [122].

A number of technical tools to monitor sterile *A. fraterculus *after released in the field, for instance sexual competitiveness [12], survival [36,46], mobility, dispersal [33-36], etc. are already available. However, research is still needed on the evaluation of sterility induced in the wild population. This is the most important parameter to be monitored according to Vreysen [123], because it provides the best evidence that the release of sterile insects causes changes in the density of the target insect.

Declaring an area to be pest free is not easy. In the case of *A. fraterculus *there is an obvious need of research on the subject. For instance Barclay, Hargrove, Clift and Meats [124] proposed a procedure involving models to deal with null trapping results and to help verify that pests are no longer present after control actions are stopped. These models depend on knowledge of the efficiency and the area of attractiveness of the traps. Above all, the most urgent need in the development of the SIT for *A. fraterculus *is finding a specific lure and designing a specific trap.

## Future research on Anastrepha fraterculus

The isolation and characterisation of specific microsatellite markers for *A. fraterculus *[106] opens a wide door to perform genetic analyses in wild populations of this pest. Valuable information on their genetic structure, dispersion patterns, and distribution of genetic variants is foreseen. In addition, these molecular markers will help in the species elucidation within the *A. fraterculus *complex (J Silva, S Lanzavecchia and others, work in progress).

These markers are also useful for exploring changes in the genetic variability suffered by a wild population of *A. fraterculus *during adaptation to artificial rearing [107]. A complete picture of the dynamics of genetic variability changes during laboratory adaptation should provide better quality control protocols for factory strains of this pest.

The extended asynapsis shown in the polytene chromosomes of F1 and F2 hybrids between two cryptic species [18] indicate an interesting venue of future research in the clarification of different entities in the *A. fraterculus *complex. This will be possible after a detailed map (presently under progress by Giardini, Zacharopoulou and others) becomes available.

Several genes involved in sex determination have been characterised in *A. fraterculus *and other *Anastrepha *species [125-128]. The elucidation of the mechanism driving early embryos to either the female or the male developmental pathway (see [129]) should be an obvious target of future research.

Key pheromone components of courtship and their roles as attractants to the two sexes have been overlooked in *A. fraterculus*. This information has several implications, such as understanding the basis of increased sexual performance after aroma exposure or protein supply, the identification of compounds that may act as sexual isolation barriers and the development of artificial attractants for mass-trapping programmes.

Genetic drift and artificial selection associated with rearing conditions could have detrimental effects on the ability of laboratory males to compete for mating in nature. This ability should be assessed in mass-rearing facilities to determine the quality of released males. A better understanding of the courtship behaviour in *A. fraterculus *could also help to understand the basis of the reproductive isolation between different morphotypes (cf. [70]).

The exact number and distribution of each entity in the *A. fraterculus *complex is not known. This knowledge would be extremely important to understand the real structure of the complex and to design effective control methods based on the SIT. About the barriers for reproduction between morphotypes, future research might address subjects as reproductive barriers at the micro-scale (cf. [105]), the role of sex pheromones and cuticle hydrocarbons in the recognition of sexes, or the role that *Wolbachia *might be playing in the build-up of reproductive barriers between members of the *A. fraterculus *complex.

## Competing interests

The authors declare that they have no competing interests.

## Aknowledgements

The careful critical reading by Don McInnis, Kostas Bourtzis and an anonymous reviewer are much appreciated. Grants from the following agencies are acknowledged: Agencia Nacional Promoción Ciencia y Técnica: through PIC 1320-2010 (to DFS). International Atomic Energy Agency: though RC 15745 (to SBL). Universidad Buenos Aires, through UBACYT 2011-2014 Nº 20020100100557 (to JCV).

This article has been published as part of *BMC Genetics *Volume 15 Supplement 2, 2014: Development and evaluation of improved strains of insect pests for SIT. The full contents of the supplement are available online at http://www.biomedcentral.com/bmcgenet/supplements/15/S2.

Publication of this supplement was funded by the International Atomic Energy Agency. The peer review process for articles published in this supplement was overseen by the Supplement Editors in accordance with BioMed Central's peer review guidelines for supplements. The Supplement Editors declare that they have no competing interests.
